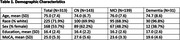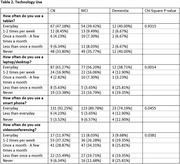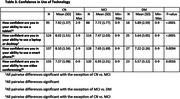# Technology Use Amongst Older Adults with and without Cognitive Impairment: Results from the ‘Validation of Video Administration of a Modified UDSv3 Cognitive Battery (VCog)’ Study

**DOI:** 10.1002/alz.093257

**Published:** 2025-01-09

**Authors:** Bonnie C. Sachs, Lauren Latham, Suzanne Craft, Sarah A. Gaussoin, Lindsay R. Clark, Kevin Duff, Sarah Tomaszewski Farias, Felicia Goldstein, Benjamin M. Hampstead, Suman Jayadev, Gregory A Jicha, Walter A. Kukull, Dawn Mechanic‐Hamilton, Judith A. Neugroschl, Kathryn V Papp, Andrew J. Saykin, Margaret Sewell, Stephen R. Rapp

**Affiliations:** ^1^ Wake Forest University School of Medicine, Winston‐Salem, NC USA; ^2^ Wake Forest School of Medicine, Winston Salem, NC USA; ^3^ University of Wisconsin‐Madison School of Medicine and Public Health, Madison, WI USA; ^4^ NIA‐Layton Aging & Alzheimer’s Disease Research Center, Portland, OR USA; ^5^ University of California, Davis, Sacramento, CA USA; ^6^ Emory University School of Medicine, Atlanta, GA USA; ^7^ Michigan Alzheimer’s Disease Research Center, Ann Arbor, MI USA; ^8^ University of Washington, Seattle, WA USA; ^9^ University of Kentucky Sanders‐Brown Center on Aging, Lexington, KY USA; ^10^ Department of Epidemiology, School of Public Health, University of Washington, Seattle, WA USA; ^11^ Penn Alzheimer’s Disease Research Center, University of Pennsylvania, Philadelphia, PA USA; ^12^ Icahn School of Medicine at Mount Sinai, New York, NY USA; ^13^ Massachusetts General Hospital, Brigham and Women’s Hospital, Harvard Medical School, Boston, MA USA; ^14^ Indiana Alzheimer’s Disease Research Center, Indianapolis, IN USA

## Abstract

**Background:**

Video interfacing is increasingly being used in research and health care. The ‘VCog’ Study seeks to determine whether remote research cognitive assessments are reliable and valid by directly comparing results from in‐person administration of a standardized cognitive battery to the same battery administered remotely by video. The study also assesses technology use and comfort amongst participants of varying levels of cognitive impairment.

**Methods:**

Participants (**Table 1**) were recruited from 12 Alzheimer’s Disease Research Centers (ADRCs). 313 ADRC enrollees who were previously classified as having normal cognition (CN), Mild Cognitive Impairment (MCI), or mild dementia were administered the Uniform Data Set v3 (UDSv3) cognitive battery remotely by video 4‐8 weeks before or after their annual in‐person UDSv3 cognitive assessments, in counterbalanced order. Participants also completed questionnaires assessing their typical use and confidence using various forms of technology.

**Results:**

More than 50% of participants reported daily‐to‐monthly use of technology, with 74‐92% of participants reporting daily smartphone use (**Table 2**). All participants reported moderate‐to‐high confidence when using technology (Range: 5.57‐8.10 on a 0‐10 scale, with 0 being ‘not confident at all’ and 10 being ‘extremely confident’). As expected, both frequency and confidence using technology varied with cognitive status; poorer cognitive status was typically associated with less usage and lower confidence (**Table 3**). Despite this, ‘dislike of video interfacing’ was not endorsed more frequently by those with cognitive impairment as the reason for study refusal (dementia = 8%; MCI = 13%; CN = 18% of total refusals).

**Conclusion:**

Technology is rapidly becoming a routine part of our lives though little is known about the relationship older adults have with it, especially those with cognitive impairment. This research indicates that among older adults of varied cognitive status, including dementia, both utilization and confidence in use are relatively high. Some group differences exist, with worse cognition being associated with less use and confidence. Despite this, persons with dementia were not likely to refuse participation because of dislike of videoconferencing. Future reports from the VCog study will report the validity and reliability of remote assessments.

**Funded by R01 AG075959**